# Creating a Machine Learning Tool to Predict Acute Kidney Injury in African American Hospitalized Patients

**DOI:** 10.3390/pharmacy10040068

**Published:** 2022-06-22

**Authors:** Sasha Pierre-Paul, Xiang S. Wang, Constance Mere, Dhakrit Rungkitwattanakul

**Affiliations:** 1 Department of Pharmacy, Howard University Hospital, Washington, DC 20059, USA; sapierre-paul@howard.edu; 2 Department of Pharmaceutical Sciences, Howard University College of Pharmacy, Washington, DC 20059, USA; xiang.wang@howard.edu; 3 Division of Nephrology, Department of Medicine, Howard University College of Medicine, Washington, DC 20059, USA; ccmere@howard.edu; 4 Department of Clinical and Administrative Pharmacy Sciences, Howard University College of Pharmacy, Washington, DC 20059, USA

**Keywords:** acute kidney injury, African Americans, machine learning

## Abstract

Machine learning (ML) has been used to build high-performance prediction models in the past without considering race. African Americans (AA) are vulnerable to acute kidney injury (AKI) at a higher eGFR level than Caucasians. AKI increases mortality, length of hospital stays, and incidence of chronic kidney disease (CKD) and end-stage renal disease (ESRD). We aimed to establish an ML-based prediction model for the early identification of AKI in hospitalized AA patients by utilizing patient-specific factors in an ML algorithm to create a predictor tool. This is a single-center, retrospective chart review. We included participants 18 years or older and admitted to an urban academic medical center. Two hundred participants were included in the study. Our ML training set provided a result of 77% accuracy for the prediction of AKI given the attributes collected. For the test set, AKI was accurately predicted in 71% of participants. The clinical significance of this model can lead to great advancements in the care of AA patients and provide practitioners avenues to optimize their therapy of choice in AAs when given AKI risk ahead of time.

## 1. Introduction

Acute kidney injury (AKI) is one of the most common medical problems found among hospitalized patients [1]. Wang et al. reported that African Americans were significantly more likely to develop AKI during hospitalization compared to their Caucasian (CS) counterparts and that AKI was independently associated with increased in-hospital mortality leading to a more than four-fold increased likelihood of death when a patient had an AKI while hospitalized [1].

Increased incidence of comorbidities as well as genetic differences contributes to AAs’ increased risk for developing AKI during hospitalization [2]. Diabetes and hypertension are the most common precipitating factors for developing chronic kidney disease. Incidence of diabetes in AAs compared to CSs is 18% vs. 9.6%, respectively [2]. Incidence of hypertension in AA compared to CS is 43.3% vs. 29.1%, respectively [2]. Along with the increased incidence of comorbidities in the AA population, genetic polymorphisms are also responsible for some variations in kidney function. It was discovered 10 years ago by Giulio Genovese that persons of African ancestry carry an apolipoprotein L1 (APOL1) genetic variation, which results in increased occurrence of AKI by as much as 15% in AAs carrying both alleles compared to CSs [3,4]. These factors compound in the AA community and contribute to the development of AKI during hospitalization and increased likelihood of death.

Aside from increased mortality and increased hospital stay, the cost burden of AKI is great on both the patient and the healthcare system in the United States. AKI is associated with an increase in hospital costs that ranges from USD 5.4 to USD 24.0 billion annually [5], with the greatest cost stemming from AKIs that require dialysis, where cost can increase anywhere from USD 11,016 to USD 42,077 per hospitalization per patient [5].

African Americans have compounded risks associated with developing AKI during hospitalization, leaving them vulnerable to poor outcomes. Early prediction of AKI in AAs is the first step in reducing the occurrence of AKI. Utilizing a tool to predict AKI can lead to shorter hospital stays, improved patient outcomes, and lower costs to the patient and the health care system. For this reason, we aimed to establish the first AKI predictor tool made specifically for AA patients admitted to the hospital utilizing machine learning (ML) software to be used for the early identification of AKI in AA patients. Successful completion of this predictor tool can lead to utilization on a larger scale with integration into electronic medical records (EMR).

Machine learning is the forthcoming advancement in patient care. This is evident in Amazon’s utilization of ML. Amazon web services use ML by utilizing health data to drive purpose-built predictor tools that help healthcare providers, payers, and pharmaceutical companies improve and accelerate diagnosis by managing population health on a global scale [6]. Amazon’s strategic use of ML on a large scale by extracting health information allows for them to forecast that improve health outcomes and decrease costs [6].

The objective of this study is to create an AKI predictor tool to accurately predict AKI in AA patients during hospital coarse based on binary and categorical information in the EMR.

## 2. Materials and Methods

This is a single-center, retrospective chart review of patients admitted to an urban academic medical center between 1 January 2021 to 1 November 2021. The EMR database was accessed to obtain participants’ information. Howard University Office of Regulatory Research Committee Institutional Review Board approved this protocol (FWA00000891).

### 2.1. AKI Definition

AKI was staged according to the 2012 Kidney Disease: Improving Global Outcomes (KDIGO) serum creatinine (SCr) criteria. Urine output was not collected hourly for each participant and was therefore not used for AKI staging. Stage 1 AKI is defined as rise in SCr 1.5–1.9 times baseline or ≥0.3 mg/dL increase over 24 h. Stage 2 AKI is defined as 2.0–2.9 times baseline SCr. Stage 3 AKI is defined as a rise in SCr 3.0 times baseline or ≥4.0 mg/dL increase over 24 h or initiation of renal replacement therapy [7]. Comparison between groups was which participant-specific factors increased the participants’ probability of developing AKI during hospitalization.

### 2.2. Participant Selection

Inclusion criteria included being of 18 years of age or older, being African American, and having a hospital length of stay greater than 48 h. Exclusion criteria included pregnancy, end-stage renal disease receiving any form of renal replacement therapy, laboratory-confirmed coronavirus disease of 2019 (COVID-19), hemodynamic support of any kind, and a hospital stay of less than 48 h.

### 2.3. Data Extraction

Participant data were retrieved from the EMR database using a randomized cohort of de-identified medical record numbers. The following information was used in this study: (1) demographic features, including sex, age, height, weight, and ethnicity; (2) comorbidities, including hyperlipidemia (HLD), type 2 diabetes (T2DM), and hypertension (HTN); (3) laboratory parameters, including SCr, GFR, WBC, drug toxicity screens, and cultures; (4) therapeutic and clinical managements, including mechanical ventilation, nephrotoxic agent used during hospital stay: vancomycin, non-steroidal anti-inflammatory drugs (NSAIDs), amphotericin b, angiotensin-converting enzyme inhibitors (ACEI) and angiotensin 2 receptor blockers (ARB), and aminoglycosides (AMG), and length of hospital stay and vasopressor use.

Data collected were categorized as categorical or binary as present or not present. Data were subsequently classified using regression, clustering, association rules mining, and visualization using Waikato Environment for Knowledge Analysis (WEKA), a data mining software platform to create a calculator. Participants included in the analysis were then divided into training and test sets. WEKA utilizes a training set of participants against a test set to determine external validity. Test and training sets do not include any duplicate participants.

### 2.4. Statistical Analysis and Machine Learning Creation

WEKA is an ML software developed by the Waikato machine learning group in the Department of Computer Science at the University of Waikato Hamilton, New Zealand, under the GNU General Public License. This internationally available workbench has established itself as a widely accepted data mining and machine learning tool in research and academia [8].

Naïve Bayes is a logistic multilayer regression tool within WEKA. Naïve Bayes uses a 10-fold cross validation to estimate the skill of the model to forecast and make predictions on unseen data based on historical results. WEKA uses the naïve Bayes theorem: P(A|B) = [P(B|A)P(A)]/P(B), which determines the probability of A happening (in this case AKI), given that B has occurred (any one of our given attributes). In this scenario, B is the evidence or the participant characteristic, and A is the hypothesis, occurrence of AKI [9].

Selection of prediction variables and model development was performed on the training set cohort only, and performance and stability were internally validated using the training set. Model performance was subsequently evaluated using the test set cohort against the rules of the training set cohort from the naïve Bayes classifier.

## 3. Results

### Baseline Characteristics

**Figure 1:**
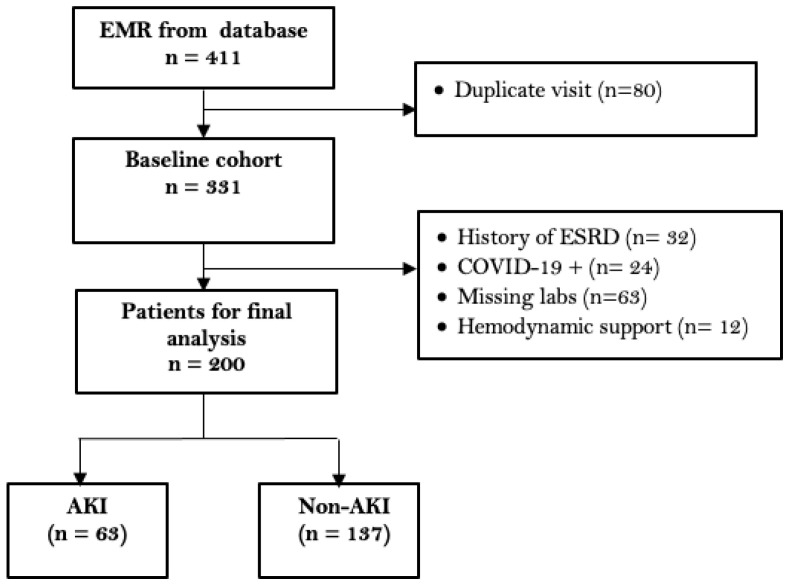
Cohort diagram.

A total of 411 EMRs were randomized to inclusion, as seen in Figure 1. A total of 200 participants were included in this analysis. A total of 80 participants were excluded from the analysis due to duplicate visits within the established protocol time frame. Thirty-two participants were excluded due to a history of end-stage renal disease (ESRD), and twenty-four participants were excluded due to laboratory-confirmed COVID-19. Sixty-three participants were excluded from the analysis due to missing labs. Missing labs included gaps in bloodwork during admission of 24 h or greater and omission of COVID-19 testing during admission.

Data collected from the 200 participants included in this analysis included: age, race, sex, comorbid conditions (HTN, HLD, and T2DM), SCr, GFR, height, weight, presence of infection, tobacco use, illicit drug use, nephrotoxic agent used during hospital stay (vancomycin, NSAID, amphotericin b, ACE-I/ARB, and AMG) and length of hospital stay. AKI occurrence per attribute is listed in Table 1, as well as occurrence per attribute in the total cohort. Of the 200 participants included in the analysis, a total of sixty-three or 31.5% of participants experienced AKI during hospitalization. Of participants that experienced AKI, 39.7% had a laboratory-confirmed infection, and 37.7% and 39.3% had HTN and HLD, respectively. Of the nephrotoxic agents used, the occurrence of AKI was greatest in participants receiving vancomycin, acyclovir, and AMG, 45.3%, 75.0%, and 50.0%, respectively.

**Figure 2:**
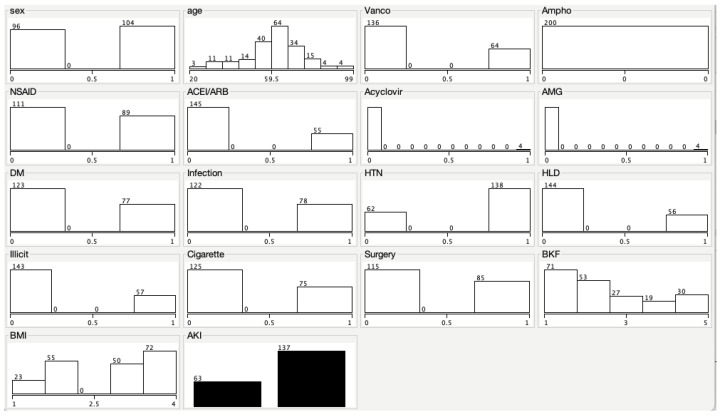
WEKA visualization output per characteristic. Sex: 0 indicates female; 1 indicates male. Age: stratified in 7.9-year increments starting at 20 years old. Vancomycin: 0 indicates participant did not receive it; 1 indicates participant received it. Amphotericin: 0 indicates participant did not receive it. NSAID: 0 indicates participant did not receive it; 1 indicates participant received it. ACEI/ARB: 0 indicates participant did not receive it; 1 indicates participant received it. Acyclovir: 0 indicates participant did not receive it; 1 indicates participant received it. AMG: 0 indicates participant did not receive it; 1 indicates participant received it. DM: 0 indicates participant did not have it; 1 indicates participant had it. Infection: 0 indicates participant did not have it; 1 indicates participant had it. HTN: 0 indicates participant did not have it; 1 indicates participant had it. HLD: 0 indicates participant did not have it; 1 indicates participant had it. Illicit: 0 indicates participant did not use illicit drugs; 1 indicates participant used illicit drugs. Cigarette: 0 indicates participant did not smoke; 1 indicates participant smoked. Surgery: 0 indicates participant did not have it; 1 indicates participant had it. BKF: stratified into 5 intervals—1 indicates GFR > 90 mL/min/1.73 m^2^; 2 indicates GFR 60–89 mL/min/1.73 m^2^; 3 indicates GFR 45–59 mL/min/1.73 m^2^; 4 indicates GFR 30–44 mL/min/1.73 m^2^; 5 indicates GFR > 30 mL/min/1.73 m^2^. BMI: stratified into 4 intervals—1 indicates BMI < 18.5 kg/m^2^; 2 indicates BMI 18.5–24.9 kg/m^2^; 3 indicates BMI 25–29.9 kg/m^2^; 4 indicates BMI > 30 g/m^2^. AKI: left indicates participant had AKI; right indicates participant did not have it.

WEKA visualization output per characteristic is illustrated in Figure 2. To the left, 0 indicates no presence of that attribute in the population, and to the right, 1 indicates yes to the presence of that attribute in the population. This follows for sex, medication, comorbidity, AKI, illicit drug use, and cigarette use. Body mass index (BMI) and beginning kidney function were stratified, and age was continuous.

**Figure 3:**
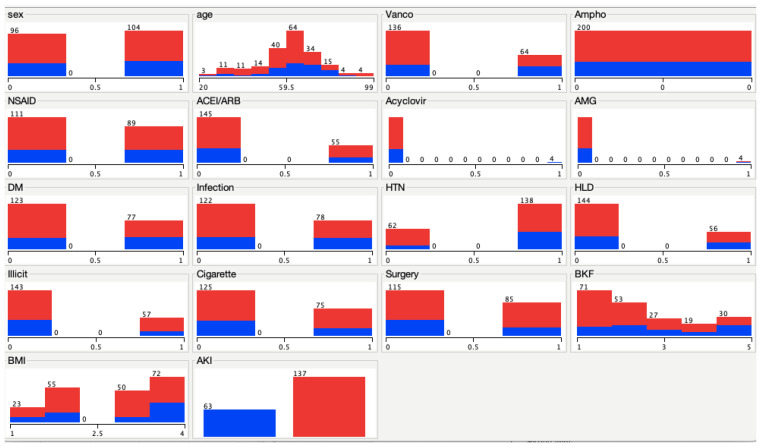
WEKA AKI visualization occurrence per characteristic. Blue indicates AKI is present per characteristic; red indicates AKI is not present per characteristic. Sex: 0 indicates female; 1 indicates male. Age: stratified in 7.9-year increments starting at 20 years old. Vancomycin: 0 indicates participant did not receive it; 1 indicates participant received it. Amphotericin: 0 indicates participant did not receive it. NSAID: 0 indicates participant did not receive it; 1 indicates participant received it. ACEI/ARB: 0 indicates participant did not receive it; 1 indicates participant received it. Acyclovir: 0 indicates participant did not receive it; 1 indicates participant received it. AMG: 0 indicates participant did not receive it; 1 indicates participant received it. DM: 0 indicates participant did not have it; 1 indicates participant had it. Infection: 0 indicates participant did not have it; 1 indicates participant had it. HTN: 0 indicates participant did not have it; 1 indicates participant had it. HLD: 0 indicates participant did not have it; 1 indicates participant had it. Illicit: 0 indicates participant did not use illicit drugs; 1 indicates participant used illicit drugs. Cigarette: 0 indicates participant did not smoke; 1 indicates participant smoked. Surgery: 0 indicates participant did not have it; 1 indicates participant had it. BKF: stratified into 5 intervals—1 indicates GFR > 90 mL/min/1.73 m^2^; 2 indicates GFR 60–89 mL/min/1.73 m^2^; 3 indicates GFR 45–59 mL/min/1.73 m^2^; 4 indicates GFR 30–44 mL/min/1.73 m^2^; 5 indicates GFR > 30 mL/min/1.73 m^2^. BMI: stratified into 4 intervals—1 indicates BMI < 18.5 kg/m^2^; 2 indicates BMI 18.5–24.9 kg/m^2^; 3 indicates BMI 25–29.9 kg/m^2^; 4 indicates BMI > 30 g/m^2^. AKI: left indicates participant had AKI; right indicates participant did not have it.

AKI occurrence per characteristic is illustrated in Figure 3. Blue indicates AKI occurred within that attribute and RED indicated no AKI occurred within that attribute.

**Figure 4:**
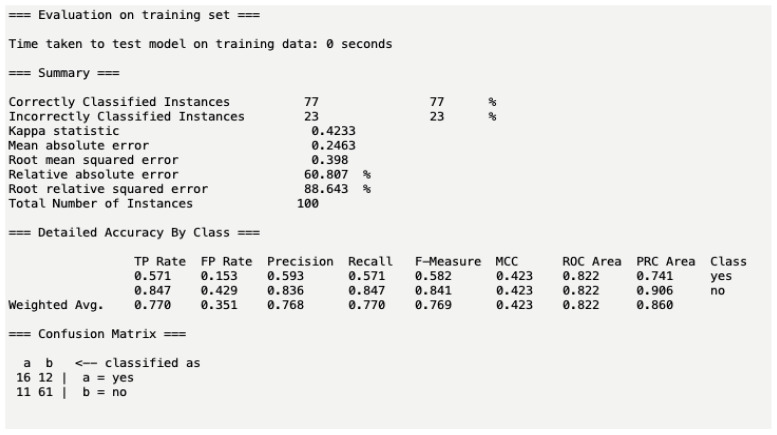
Training set model performance. Definitions [10,11,12,13,14,15,16,17,18,19]: Correctly classified instances—the sum of true positives and true negatives. Incorrectly classified instances—the sum of false positives and false negatives. Kappa statistic—values ≤ 0 as indicating no agreement and 0.01–0.20 as none to slight, 0.21–0.40 as fair, 0.41–0.60 as moderate, 0.61–0.80 as substantial, and 0.81–1.00 as almost perfect agreement. Mean absolute error—the magnitude of difference between the prediction of observation and the true value of that observation. Means absolute percentage error (MAPE) < 10% is excellent and MAPE < 20% is good. Root mean square error residuals are a measure of how far from the regression line data points are; RMSE is a measure of how spread out these residuals are. In other words, it tells you how concentrated the data are around the line of best fit. Relative absolute error—indicates how well a model performs relative to the average of the true values. When the RRSE is <than one, the model performs better than the simple model. The lower the RRSE, the better the model. TP rate—true-positive cases. FP rate—false-positive cases. Precision—the ability of a classification model to identify only the relevant data points. Mathematically, precision is the number of true positives divided by the number of true positives plus the number of false positives. Recall—the ability of a model to find all the relevant cases within a data set. Mathematically, we define recall as the number of true positives divided by the number of true positives plus the number of false negatives. F-Measure considers both precision and recall to compute. F1 score reaches its best value at 1 and worst value at 0. MCC—a correlation of: C = 1 indicates perfect agreement, C = 0 is expected for a prediction no better than random, and C = −1 indicates total disagreement between prediction and observation. ROC area—an AUC of 0.5 suggests no discrimination, from 0.7 to 0.8 is considered acceptable, from 0.8 to 0.9 is considered excellent, and more than 0.9 is considered outstanding. PRC area—shows the relationship between precision (=positive predictive value) and recall (=sensitivity) for every possible cut-off. Average precision ranges from the frequency of positive examples from 0.5 (for balanced data) to 1.0 (perfect model).

In the training set, cross validation of data was performed using naïve Bayes on randomly selected training data (*n* = 100). This training model performed well, with a 77% accuracy in correctly classified instances, as seen in Figure 4. This means that this test set will accurately predict AKI 77% of the time when given these attributes. The mean absolute error percentage was 24.6%, which indicates a good fit of this model to predict AKI versus the actual occurrence of AKI. The AUC ROC curve of 0.860 also indicates that this training set yields excellent performance with a classifier model, lending credibility to this model to predict AKI correctly in the test set.

**Figure 5:**
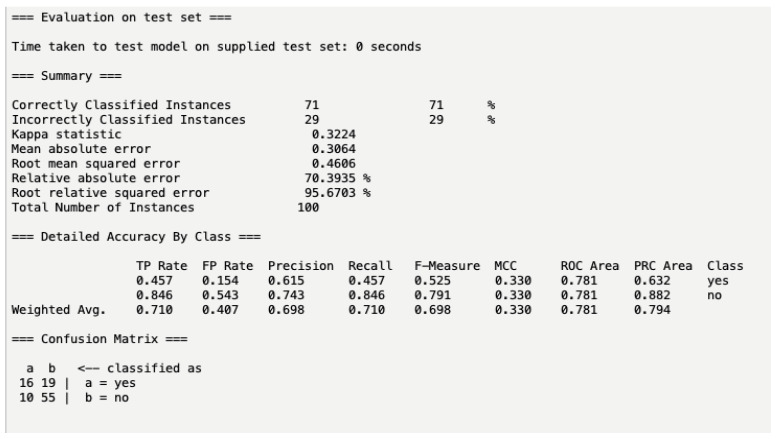
Test set model performance. Definitions [10,11,12,13,14,15,16,17,18,19]: Correctly classified instances—the sum of true positives and true negatives. Incorrectly classified instances—the sum of false positives and false negatives. Kappa statistic—values ≤ 0 as indicating no agreement and 0.01–0.20 as none to slight, 0.21–0.40 as fair, 0.41–0.60 as moderate, 0.61–0.80 as substantial, and 0.81–1.00 as almost perfect agreement. Mean absolute error—the magnitude of difference between the prediction of an observation and the true value of that observation. Means absolute percentage error (MAPE) < 10% is excellent and MAPE < 20% is good. Root mean square error residuals are a measure of how far from the regression line data points are; RMSE is a measure of how spread out these residuals are. In other words, it tells you how concentrated the data are around the line of best fit. Relative absolute error—indicates how well a model performs relative to the average of the true values. When the RRSE is <than one, the model performs better than the simple model. The lower the RRSE, the better the model. TP Rate—true-positive cases. FP Rate—false-positive cases. Precision—the ability of a classification model to identify only the relevant data points. Mathematically, precision is the number of true positives divided by the number of true positives plus the number of false positives. Recall—the ability of a model to find all the relevant cases within a data set. Mathematically, we define recall as the number of true positives divided by the number of true positives plus the number of false negatives. F-Measure considers both precision and recall to compute the score. F1 score reaches its best value at 1 and worst value at 0. MCC—a correlation of: C = 1 indicates perfect agreement, C = 0 is expected for a prediction no better than random, and C = −1 indicates total disagreement between prediction and observation. ROC area—an AUC of 0.5 suggests no discrimination, from 0.7 to 0.8 is considered acceptable, from 0.8 to 0.9 is considered excellent, and more than 0.9 is considered outstanding. PRC area—shows the relationship between precision (=positive predictive value) and recall (=sensitivity) for every possible cut-off. Average precision ranges from the frequency of positive examples from 0.5 (for balanced data) to 1.0 (perfect model).

In the test set, the validation was performed using naïve Bayes models built from data (*n* = 100). The participants in the test data (*n* = 100) did not include any identical participants from the training data. This test set performed well against the training model, with a 71% accuracy in correctly classified instances, as seen in Figure 5. This means that this test set will accurately predict AKI 71% of the time when given these attributes. The mean absolute error percentage was 30.6%, which indicates a good fit of this model to predict AKI versus the actual occurrence of AKI. The area under ROC curve is 0.781, indicating that this test set yields a good performance as a classifier model, differing slightly from the training set.

## 4. Discussion

Factors that contribute to AAs’ increased risk for developing AKI during hospitalization include increased incidence of comorbidities as well as genetic differences [3]. Concerning comorbidities, diabetes and hypertension are the most common precipitating factors for developing CKD. African Americans have compounded risk associated with developing AKI during hospitalization, leaving them vulnerable to poor outcomes. Early prediction of AKI in AAs is the first step in reducing the occurrence of AKI. For this reason, we aimed to establish the first AKI predictor tool made specifically for AA participants admitted to the hospital utilizing ML to be used for early identification of AKI in AA participants.

Our model accurately predicted true positives and true negatives of AKI more frequently than several available published models [20,21]. Our predictor tool provided a ROC curve of 0.860 (AUC) within our training set, indicating excellent performance as a classifier model, as seen in Figure 4. Compared with previous reports of AKI prediction, our test set provided a superior prediction of AKI in the test set population. In the study led by a group of Chinese investigators, they utilized ML to predict AKI in intensive care participants and found their tool to yield a ROC curve of 0.817 in AUC [20]. Our model predicted AKI correctly more than their prediction model. Yue et al.’s predictor model also provided good predictive accuracy in terms of discrimination and calibration, with recall and F1 scores of 0.852 and 0.895, respectively [20]. This again compared to our predictor tool, which has the recall and F1 scores of 0.770 and 0.769, respectively. In Yue’s study, they aimed to predict AKI among sepsis patients. Sepsis-induced AKI can occur in several factors, including the timing of antibiotic administration, antibiotic selection, drug and pathogen resistance pattern, and fluid administration. Yue et al. did not include the nuances of several etiologies of sepsis in their model. This could explain their lower AUC ROC compared to our study.

The study led by Yue et al. does not offer the same clinical utility in the AA population as this study, as they did not include a diverse participant population. The comparator calculator measured for ethnicity, where white participants accounted for 75.3% of participants and black participants accounted for just 8.2% [20]. The race was not found to have a statistically significant effect in causing AKI. Given the increased occurrence of AKI in AAs, it is imperative that AAs hold a clinically significant part in studies such as these. In the 2016 cross-sectional survey of the National Hospital Discharge Survey of 276,138 participants by Mathioudakis et al., it was found that black patients had 50% higher odds of having AKI while inpatient. It was also found that black patients were more likely to have comorbid conditions that increase the risk of AKI during hospitalization, including sepsis (2.7% vs. 2.2%, *p* < 0.001) and CKD (5.0% vs. 4.0%, *p < 0.001*) [22]. This further indicates the need for a predictor tool, such as the one validated in this study, to provide comprehensive care for all patients.

Patient characteristics have shown that what increases the risk for developing kidney disease were also principal characteristics in patients that develop AKI inpatient. As seen in Table 1, infection and diabetes accounted for the largest amount of AKI per characteristic, 39.7% and 41.6%, respectively, aside from nephrotoxic agents. As patients battle infection, severe sepsis, for example, AKI precipitates as a result of ischemia. End organ kidney damage can often lead to metabolic derangement that results in increased length of hospital stay and increased mortality. Therefore, accounting for infection in predictive models is vital. If we can predict AKI in these patients and optimize therapy choice before AKI precipitates, we can decrease renal exposure and decrease hospital length of stay and costs.

Our cohort included only 100 participants for the classification of our model compared to the 3176 participants included in the study by Yue et al. [20]. The amount of data required for ML to correctly classify prediction is summed up by the rule of ten. The rule of ten states that ten times the number of parameters, or degrees of freedom, in the model were needed to allow for ML to correctly classify training data [23]. For this reason, the cohort size used in their analysis is a strength and limitation of our study. Nineteen parameters were used in our study, and while a total of 200 participants were included in this analysis, the split into training set and test set failed to reach the hypothesized ideal sample size. The comparator predictor tool had 56 parameters with greater than 10 times per parameter number of participants included in the analysis. Regardless, our model still showed 77% accuracy and AUC ROC of 0.86, signifying that it is still powerful and accurate.

KDIGO guidelines for the management of AKI suggested using SCr and urine output for screening. Patients with urine output of less than 0.5 mL/kg/h for 6–12 h can be classified as having AKI regardless of their SCr values. However, in our study, urine output was not collected appropriately on all included participants and was therefore not used to stage the AKI. This may limit the diagnosis of AKI in patients who did not have acute rises in SCr but had decreased urine outputs [7].

What is next for this study is the development of a website or application interface for practitioners to use to input patient attributes. Development of this model and its algorithm into a calculator for ease of use would allow for further external validation from third parties. This is a critical step in taking this research nationwide, with the eventual goal of being immersed in EMRs. This would allow for seamless integration and predictive ability with a possible added benefit of therapy choice direction.

## 5. Conclusions

Our AKI predictor tool performed well, with a 77% accuracy in correctly classified instances in correctly predicting AKI in the training set. The clinical significance of this model can lead to great advancements in the care of AA patients and provide practitioners avenues to optimize their therapy of choice in AAs when given AKI risk ahead of time. Development of this model and its algorithm into a calculator for ease of use would allow for further external validation from third parties.

## Figures and Tables

**Table 1:** Participant characteristics.

Characteristic	N = 200 (%)	AKI (% per Characteristic)	No AKI (% per Characteristic)
Gender	Male: 104 (52%) Female: 96 (48%)	Male: 34 (32.7%) Female: 29 (30.2%)	Male: 70 (67.3%) Female: 67 (69.8%)
Age	Mean: 60.94 years	-	-
BMI	Mean range: 18.5–24.9 kg/m^2^	-	-
Baseline kidney Function	Mean GFR: 45–89 mL/min/1.73 m^2^	-	-
Presence of infection	78 (39.0%)	31 (39.7%)	47 (60.3%)
Hypertension	138 (69.0%)	52 (37.7%)	86 (62.3%)
Hyperlipidemia	56 (28.0%)	22 (39.3%)	34 (60.7%)
T2DM	77 (38.5%)	32 (41.6%)	45 (58.4%)
Vancomycin	64 (32%)	29 (45.3%)	35 (54.7%)
NSAID	89 (44.5%)	31 (34.8%)	58 (65.2%)
ACEI/ARB	55 (27.5%)	17 (30.9%)	38 (69.1%)
Acyclovir	4 (2.0%)	3 (75.0%)	1 (25.0%)
Aminoglycoside	4 (2.0%)	2 (50.0%)	1 (25.0%)
Amphotericin B	0 (0%)	0 (0%)	0 (0%)
Illicit drug use	57 (28.5%)	14 (24.6%)	43 (75.4%)
Cigarette use	75 (37.5%)	21 (28.0%)	54 (72.0%)
Surgery	85 (42.5%)	22 (25.9%)	63 (74.1%)
AKI	63 (31.5%)	-	137 (68.5%)

Abbreviation: BMI—body mass index; T2DM—type 2 diabetes mellitus; NSAIDs—non-steroidal anti-inflammatory drugs; AKI—acute kidney injury.

## References

[1] Wang H.E., Muntner P., Chertow G.M., Warnock D.G. (2012). Acute kidney injury and mortality in hospitalized patients. Am. J. Nephrol..

[2] Dummer P.D., Limou S., Rosenberg A.Z., Heymann J., Nelson G., Winkler C.A., Kopp J.B. (2015). APOL1 kidney disease risk variants: An evolving landscape. Semin Nephrol..

[3] Laster M., Shen J.I., Norris K.C. (2018). Kidney disease among African Americans: A population perspective. Am. J. Kidney Dis..

[4] Kolata G. Targeting the uneven burden of kidney disease on black Americans. The New York Times.

[5] Silver S.A., Chertow G.M. (2017). The economic consequences of acute kidney injury. Nephron.

[6] Amazon.com. https://aws.amazon.com/machine-learning/.

[7] Khwaja A. (2012). KDIGO clinical practice guidelines for acute kidney injury. Nephron Clin. Pract..

[8] Hall M., Frank E., Holmes G., Pfahringer B., Reutemann P., Witten I.H. (2009). The WEKA data mining software: An update. SIGKDD Explor..

[9] Gandhi R. Naive Bayes classifier. Towards Data Science. https://towardsdatascience.com/naive-bayes-classifier-81d512f50a7c.

[10] McHugh M.L. (2012). Interrater reliability: The kappa statistic. Biochem. Med..

[11] Mean Absolute Error. C3 AI. https://c3.ai/glossary/data-science/mean-absolute-error/.

[12] Coding Prof. 3 Ways to Calculate the Root Relative Squared Error (RRSE) in R. CodingProf.com. https://www.codingprof.com/3-ways-to-calculate-the-root-relative-squared-error-rrse-in-r/.

[13] RMSE: Root Mean Square Error. Statistics How To. https://www.statisticshowto.com/probability-and-statistics/regression-analysis/rmse-root-mean-square-error/.

[14] Koehrsen W. When Accuracy Isn’t Enough, Use Precision and Recall to Evaluate Your Classification Model. Built in. https://builtin.com/data-science/precision-and-recall.

[15] Precision, Recall and Correctly Classified Instances. Stack Overflow. https://stackoverflow.com/questions/25349841/precision-recall-and-correctly-classified-instances.

[16] Get Your Guide. What is a Good F1 Score?—Inside Get Your Guide. https://inside.getyourguide.com/blog/2020/9/30/what-makes-a-good-f1-score.

[17] Towardsdatascience.com. https://towardsdatascience.com/the-best-classification-metric-youve-never-heard-of-the-matthews-correlation-coefficient-3bf50a2f3e9.

[18] Mandrekar J.N. (2010). Receiver operating characteristic curve in diagnostic test assessment. J. Thorac. Oncol..

[19] Precision-Recall Curves—What are They and How are They Used? Acutecaretesting.org. https://acutecaretesting.org/en/articles/precision-recall-curves-what-are-they-and-how-are-they-used.

[20] Yue S., Li S., Huang X., Liu J., Hou X., Zhao Y., Niu D., Wang Y., Tan W., Wu J. (2022). Machine learning for the prediction of acute kidney injury in patients with sepsis. J. Transl. Med..

[21] Deng Y.H., Luo X.Q., Yan P., Zhang N.Y., Liu Y., Duan S.B. (2022). Outcome prediction for acute kidney injury among hospitalized children via eXtreme Gradient Boosting algorithm. Sci. Rep..

[22] Mathioudakis N.N., Giles M., Yeh H.C., Haywood C., Greer R.C., Golden S.H. (2016). Racial differences in acute kidney injury of hospitalized adults with diabetes. J. Diabetes Complicat..

[23] Melvin R.L. Sample Size in Machine Learning and Artificial Intelligence. Uab.edu. https://sites.uab.edu/periop-datascience/2021/06/28/sample-size-in-machine-learning-and-artificial-intelligence/.

